# Smallpox lesion characterization in placebo-treated and tecovirimat-treated macaques using traditional and novel methods

**DOI:** 10.1371/journal.ppat.1012007

**Published:** 2024-02-22

**Authors:** Todd M. Bell, Paul Facemire, Jeremy J. Bearss, Jo Lynne Raymond, Jennifer Chapman, Xiankun Zeng, Joshua D. Shamblin, Janice A. Williams, Douglas W. Grosenbach, Dennis E. Hruby, Inger K. Damon, Arthur J. Goff, Eric M. Mucker

**Affiliations:** 1 U.S. Army Medical Research Institute of Infectious Diseases (USAMRIID), Frederick, Maryland, United States of America; 2 SIGA Technologies, Inc., Corvallis, Oregon, United States of America; 3 Poxvirus and Rabies Branch, Centers for Disease Control and Prevention (CDC) Atlanta, Georgia, United States of America; Centro de Biologia Molecular Severo Ochoa, SPAIN

## Abstract

Smallpox was the most rampant infectious disease killer of the 20^th^ century, yet much remains unknown about the pathogenesis of the variola virus. Using archived tissue from a study conducted at the Centers for Disease Control and Prevention we characterized pathology in 18 cynomolgus macaques intravenously infected with the Harper strain of variola virus. Six macaques were placebo-treated controls, six were tecovirimat-treated beginning at 2 days post-infection, and six were tecovirimat-treated beginning at 4 days post-infection. All macaques were treated daily until day 17. Archived tissues were interrogated using immunohistochemistry, *in situ* hybridization, immunofluorescence, and electron microscopy. Gross lesions in three placebo-treated animals that succumbed to infection primarily consisted of cutaneous vesicles, pustules, or crusts with lymphadenopathy. The only gross lesions noted at the conclusion of the study in the three surviving placebo-treated and the Day 4 treated animals consisted of resolving cutaneous pox lesions. No gross lesions attributable to poxviral infection were present in the Day 2 treated macaques. Histologic lesions in three placebo-treated macaques that succumbed to infection consisted of proliferative and necrotizing dermatitis with intracytoplasmic inclusion bodies and lymphoid depletion. The only notable histologic lesion in the Day 4 treated macaques was resolving dermatitis; no notable lesions were seen in the Day 2 treated macaques. Variola virus was detected in all three placebo-treated animals that succumbed to infection prior to the study’s conclusion by all utilized methods (IHC, ISH, IFA, EM). None of the three placebo-treated animals that survived to the end of the study nor the animals in the two tecovirimat treatment groups showed evidence of variola virus by these methods. Our findings further characterize variola lesions in the macaque model and describe new molecular methods for variola detection.

## Author summary

The worldwide 2022 mpox outbreak renewed research interest in defining and countering threats posed by orthopoxviruses, including smallpox. In the absence of optimized prevention and treatment strategies, natural re-emergence of smallpox or its use as a bioweapon released into unvaccinated populations could result in avoidable illnesses and deaths. Here we applied recently developed techniques to study the relationship between smallpox lesion development and the fate of virus in three groups of animals exposed to smallpox virus: those treated with the FDA-approved antiviral tecovirimat 2 or 4 days after infection and a placebo-treated group. We were able to characterize the development of lesions at gross and microscopic levels and describe new molecular methods for studying the distribution and persistence of variola virus (VARV) in tissue samples that were collected from the three treatment groups. These findings can be used to fine-tune the timing of administering tecovirimat, other antivirals, and vaccines, as well as help characterize the performance of in-development orthopoxvirus countermeasures.

## Introduction

Smallpox was successfully eradicated worldwide by 1980 through an intensive surveillance, vaccination, and containment program led by the World Health Organization [1–4]. Because of the large-scale success of this unprecedented eradication effort, ongoing vaccination campaigns were discontinued for the general public creating a vacuum of protection against smallpox and other zoonotic poxviral infections.

In the weeks immediately following the 9/11 terrorist attacks, the anthrax letter attacks focused U.S. government attention on national security and existing biodefense gaps, including the gap in poxvirus immunity [5–8]. To address this gap, the U.S. government funded vaccine and antiviral development for biodefense purposes [9–11]. Animal models of smallpox disease as well as next generation vaccines and antivirals were the result [12–17], including tecovirimat (formerly ST-246), an antiviral that is specific for orthopoxviruses. The mechanism of action of this antiviral is inhibition of the VP37 protein preventing proper formation of enveloped virions (EV)[18,19]. It was FDA approved through use of the Animal Rule and was a beneficiary of post-9/11 drug development funding [13,14,20–26].

Using archived tissues from a study conducted at the Centers for Disease Control and Prevention, we endeavored to describe lesions in placebo-treated macaques to further characterize the model and describe lesion mitigation in tecovirimat-treated macaques. We also wanted to determine if viral antigen was detectable 28 days postinfection in either placebo-treated or tecovirimat-treated macaques, indicating viral persistence. Viral persistence amongst other high consequence pathogens has been an active area of research at the U.S. Army Medical Research Institute of Infectious Diseases (USAMRIID) over the past decade [27–32], and with our rich archive of formalin-fixed, paraffin-embedded (FFPE) tissues infected with high consequence pathogens, our goal was to add to the growing database of knowledge regarding viral persistence, particularly in immune privileged sites in this smallpox model.

Much of what we know about smallpox is based on data gathered prior to the eradication of smallpox and the last reported natural human case in 1977. This was prior to the bulk of the biotech revolution, thus limiting our knowledge of the molecular basis of smallpox. Over the past ten years, new and improved orthopoxvirus-specific molecular and immunodiagnostic tools have been developed at USAMRIID to better understand viral pathogenesis, allowing more sensitive detection of viral antigen and viral nucleic acid in tissues.

This study applies some of these new methods to archived FFPE tissues, collected as part of a previous study and stored at USAMRIID, to describe the pathology of placebo-treated versus tecovirimat-treated animals [23]. RNA *in situ* hybridization, immunofluorescence assays, and electron microscopic examination from select target tissues in animals from each treatment group were performed. Results are presented along with observations made during gross necropsies performed on all animals.

Due to the paucity of published pathology data on NHP models of smallpox disease, we endeavored to better characterize the gross lesions and histopathologic findings for this disease model to include a characterization of lesions between placebo-treated and tecovirimat-treated groups. Additionally, we wanted to determine the utility of newly developed molecular, immunodiagnostic, and ultrastructural methods in this characterization and to determine if virus remained in any tissues at the end of the study. We were able to use this arsenal of techniques to further characterize the model and show a lack of virus across all treatment groups and tissues examined.

## Results

### Gross pathology

Three of six placebo-treated animals survived to the end of the study (28–30 days post-exposure) and three met clinical endpoint criteria between days 13 and 14 and were humanely euthanized (See survival curve from Mucker et al[23]. All 12 tecovirimat-treated NHPs survived exposure to the end of the study. (See Table 1). Skin lesions observed, maximum viremia, time to maximum viremia, and survival by tecovirimat treatment day are listed in Table 1 and are from previously published data (ref [23]).

**Table 1:** Skin lesions observed, maximum viremia, time to maximum viremia, and survival by tecovirimat treatment day.

Nonhuman Primate Number	Treatment	Skin Lesions		Viremia (gen/mL)		Time To Maximum Viremia (Days)		Outcome* (Day Euthanized)
	Day	Maximum Observed	Group Average	Maximum Observed	Group Average	Maximum Viremia	Group Average	
3	2	655	269	2.83E+06	1.03E+06	4	3.2	S(29)
6	2	46		3.73E+05		4		S(28)
8	2	77		3.11E+04		2		S(29)
11	2	393		1.25E+06		2		S (30)
12	2	131		1.31E+06		4		S (28)
18	2	310		4.07E+05		4		S (30)
1	4	419	482	1.58E+05	4.32E+06	2	4.7	S(30)
5	4	63		2.44E+06		4		S(28)
10	4	335		1.73E+05		4		S(28)
14	4	870		2.12E+07		7		S(28)
16	4	917		7.63E+05		7		S(29)
17	4	286		1.16E+06		4		S(28)
2	Placebo	1329	1479	6.47E+06	7.68E+06	7	7	S(29)
7	Placebo	1682		6.48E+06		7		S(29)
15	Placebo	514		4.21E+05		7		S(29)
4	Placebo	1873		6.44E+06		7		E(14)
9	Placebo	1575		9.67E+06		7		E(14)
13	Placebo	1900		1.66E+07		7		E(13)

* S, Survived until end of study; E, met clinical criteria for euthanasia previous to end of study

Note: End of study necropsies were dispersed over 3 days due to the extreme amount of time and effort it took to do the NHP necropsies and sample collections in BSL-4. Data for the number of skin lesions observed, maximum viremia, time to maximum viremia, and survival is from previously published data (ref [23]).

**Fig 1:**
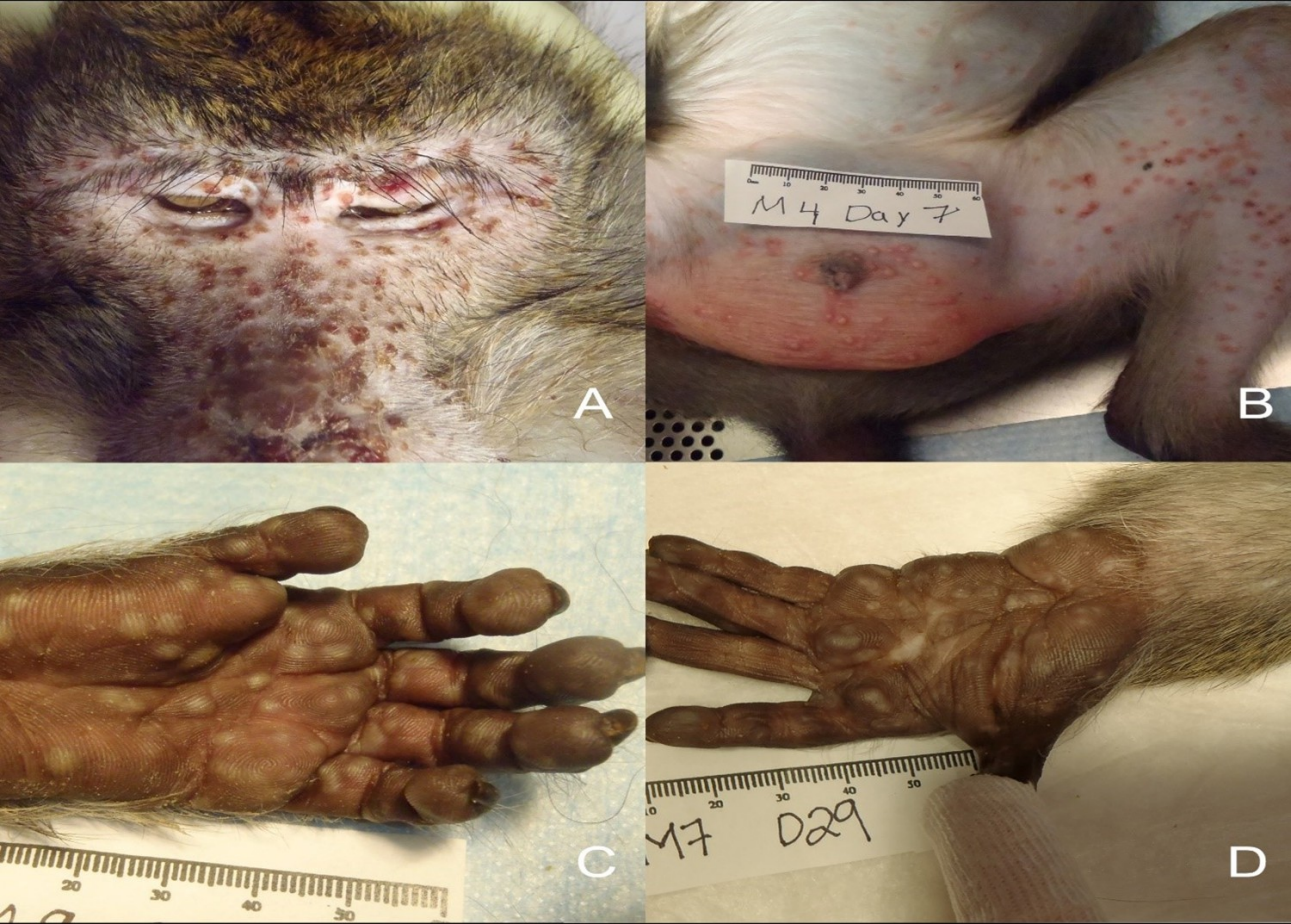
Variola-infected, placebo treated NHPs. Multifocal proliferative and necrotizing dermatitis (pox lesions). A) Facial skin (13 days post infection); B) Abdominal skin, left inner thigh, and scrotum (7 days post infection); C) Glabrous skin, left palm: multifocal proliferative dermatitis (7 days post infection); D) Glabrous skin, left palm: multifocal healed lesions with depigmentation (29 days post infection).

The three NHPs that did not survive to the end of the study had gross lesions consistent with those previously described in the ordinary smallpox model of infection (33). The following gross findings were noted in these three animals (See Table 2): cutaneous vesicles, pustules, crusts and/or scars (Fig 1, includes images of those that met criteria and those that survived until the end of the study) (3/3); enlarged lymph nodes (3/3); pale, raised pleural lesion(s) or pleural adhesions (2/3); mottled red lung lobes (1/3); ulceration within the oral cavity (3/3); dark red, enlarged spleen (3/3); esophageal hemorrhagic foci/ulcers (1/3); gastrointestinal mucosal petechial hemorrhage or erosions (3/3).

The remaining three placebo-treated animals had gross lesions limited to resolving (healing) poxviral lesions on the feet and/or hands, with 2 also having grossly enlarged spleens and one having enlarged lymph nodes at the study’s conclusion. These healing skin lesions were pale, circular, poorly pigmented areas on the palms and footpads. (See Table 2 and Fig 1).

**Table 2:** Gross findings by tecovirimat treatment group.

	Nonhuman Primate Number																	
	Placebo						Day 2 Treatment						Day 4 Treatment					
Observed Lesions	9*	13*	4*	7	2	15	11	18	3	8	6	12	1	16	14	17	5	10
Cutaneous vesicles, pustules, crusts &/or scars	X	X	X	—	—	—	—	—	—	—	—	—	—	—	—	—	—	—
Enlarged lymph nodes	X	X	X	—	X	—	—	—	—	X	—	—	—	—	X	X	—	—
Pale, raised pleural lesion(s) or pleural adhesions	X	X	—	—	—	—	—	—	X	X	—	—	—	—	X	—	—	—
Mottled red lung lobes	—	—	X	—	—	—	—	—	—	—	—	—	—	—	—	—	—	—
Oral cavity ulcers	X	X	X	—	—	—	—	—	—	—	—	—	—	—	—	—	—	—
Enlarged spleen	X	X	X	—	X	X	X	—	X	X	—	—	X	—	—	X	—	—
Esophageal hemorrhagic foci/ulcers	X	—	—	—	—	—	—	—	—	—	—	—	—	—	—	—	—	—
Gastrointestinal mucosal petechial hemorrhage or erosions	X	X	X	—	—	—	—	—	—	—	—	—	—	—	—	—	—	—
Resolving Pox lesions on feet or hands	—	—	—	X	X	X	—	—	—	—	—	—	—	—	X	X	—	—

X = finding noted;— = no finding

*Euazed prior to end of study because study endpoint met; all other animals survived to end of study

Among the 12 tecovirimat-treated NHPs, only 2 treated animals, those from the Day 4 tecovirimat group [14,17] had notable lesions consisting of resolving poxviral lesions of the skin; other findings in these animals were related to an expected immunologic response to infection with enlargement of the lymph nodes and spleen, and non-specific inflammation with respect to the pleural adhesions. The healing skin lesions in these 2 NHPs were consistent with resolving poxviral infection as described above and noted in Table 2.

### Histology, immunohistochemistry, In situ hybridization, immunofluorescence, and electron microscopy

**Fig 2:**
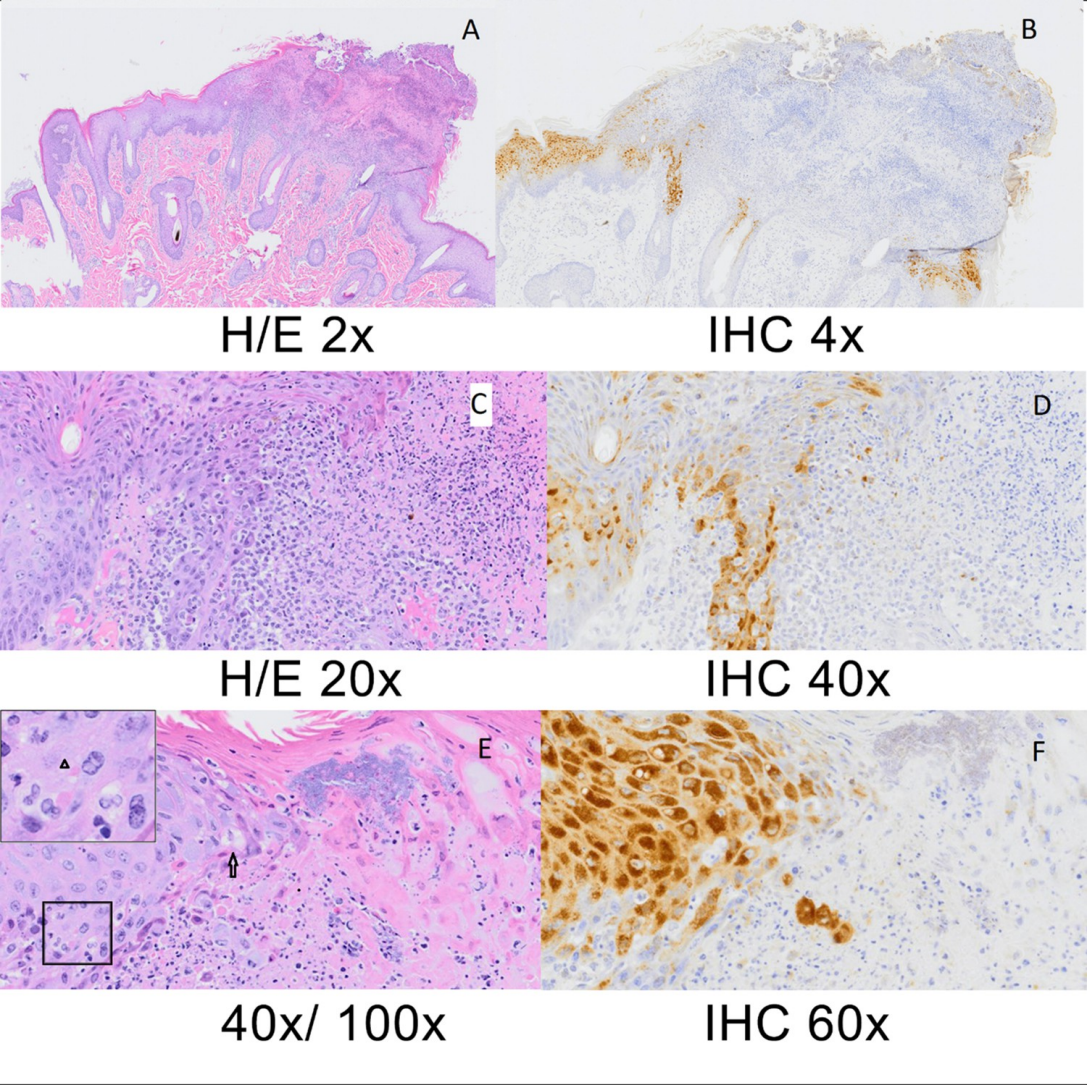
Variola-infected, placebo treated NHP x days post infection. **Haired skin, pox lesions.** A: (H&E, 4x) Proliferative and necrotizing dermatitis. B: (IHC, 4x) Viral antigen is present within epithelial cells at the edge of the pox lesion. C: (H&E 20x) Higher magnification of A, Necrotic debris and superficial serocellular crust. D: (IHC, 40x) Viral antigen in epithelial cells. E: (40x/100x) Higher magnification of A, Pyknotic and karyorrhectic (necrotic) debris with intracytoplasmic inclusions (arrowhead; inset, higher magnification of black box) and ballooning degeneration (arrow) as well as surface colonies of bacteria. F: (IHC, 60x) Viral antigen in epithelial cells.

In the placebo-treated group, the 3 macaques that succumbed prior to the end of the study had histologic lesions and positive immunohistochemical findings consistent with VARV infection. The histopathology included proliferative and/or necrotizing dermatitis (3/3); proliferative and/or necrotizing glossitis (1/3); tonsillitis (2/3); proliferative and/or necrotizing dermatitis within the nares (2/3) and lip (3/3); lymphadenitis (3/3); lymphoid depletion within the spleen (2/3); necrotizing gastritis (1/3); and necrotizing enteritis (2/3). (See Table 3 and Fig 2).

**Table 3:** Summary of histologic findings for placebo treated NHPs that met study endpoint before end of study.

Location Histologic Findings	Nonhuman Primate Number		
	9	13	4
**Haired skin** Dermatitis, proliferative and/or necrotizing	2	4	2
**Tongue** Glossitis, proliferative and/or necrotizing	2	—	—
**Tonsil** Tonsillitis +/- Lymphoid changes	—	2	2
**Nares** Dermatitis, proliferative and/or necrotizing	3	—	3
**Lip** Dermatitis, proliferative and/or necrotizing	2	2	3
**Lymph node(s)** Lymphadenitis	4	2	3
**Spleen** Lymphoid depletion	3	2	—
**Stomach** Gastritis, necrotizing	—	3	—
**Intestine** Enteritis, necrotizing	—	3	4

Severity scores: 1 = Minimal, 2 = Mild, 3 = Moderate, 4 = Severe;— = no finding

NOTE: No significant lesions were seen in the following organs: submandibular salivary gland, larynx, esophagus, trachea, thyroid gland, parathyroid gland, testis, liver, spleen, adrenal glands, pituitary gland, kidneys, urinary bladder, prostate gland, heart, lung, skeletal muscle sciatic nerve, brachial plexus, brain, eyes, and femoral bone marrow.

All three of these animals had multiple epithelial surfaces that were multifocally IHC positive to include haired skin (3/3), tongue (1/3), lip (2/3), nares (1/3), tonsil (2/3), jejunum (1/3) and ileocecal junction (1/3). See Table 5 and Fig 2 for detailed IHC results.

**Fig 3:**
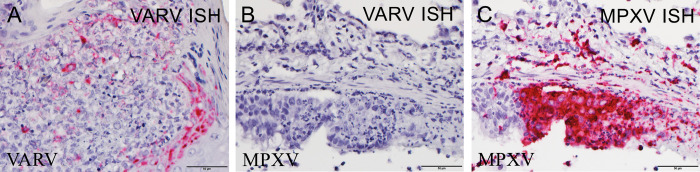
The specificity of variola virus-specific RNA in situ hybridization (ISH). A) variola virus (VARV) RNA was detected in the tongue tissue of a cynomolgus macaque, (placebo-treated #9) that succumbed to intravenous exposure of VARV using RNA ISH. B-C.) VARV RNA was undetectable in the lung tissue of a cynomolgus macaque that succumbed to aerosol exposure MPX virus using VARV-specific ISH (B), in which MPXV RNA was readily detected by MPXV-specific ISH (C).

**Fig 4:**
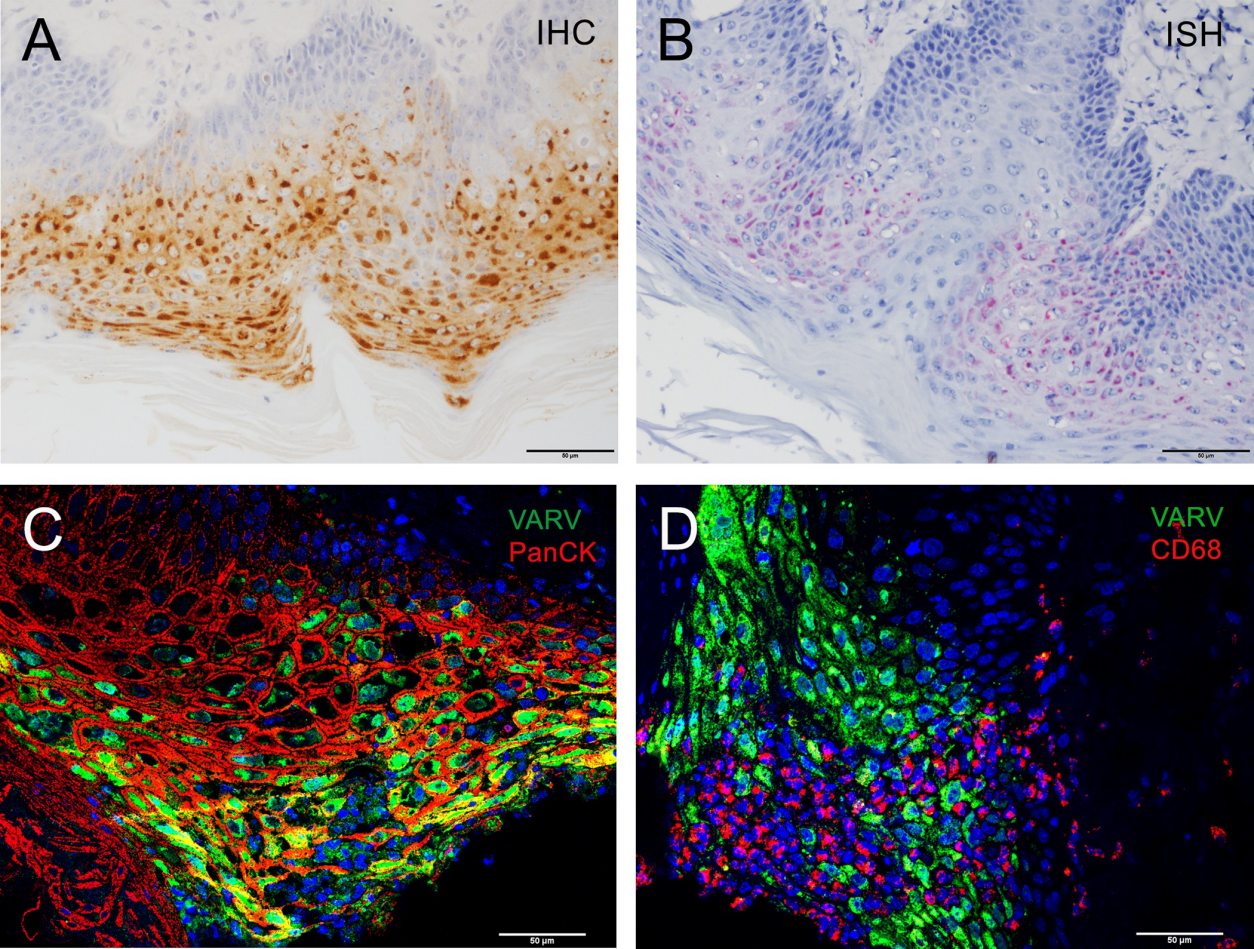
Detection of variola virus infection using molecular techniques. A-B) Variola virus (VARV) antigen (brown in A) and RNA (red in B) were detected in the haired skin of NHP #13, a placebo-treated cynomolgus macaque that succumbed to VARV exposure; immunohistochemistry (IHC) and RNA in situ hybridization (ISH), respectively. C-D) Immunofluorescence staining demonstrates VARV viral antigen (green in C and D) was primarily detected in epithelial cells (red, C) of NHP #4, a placebo-treated cynomolgus macaque, stained by anti-pan-cytokeratin (PanCK) antibody, but not in macrophages stained by anti-C68 antibody (red, D). Nuclei were counterstained blue with 4’,6-diamidino-2-phenylindole (DAPI).

To further validate the VARV infection, we developed an RNA *in situ* hybridization (ISH) assay to detect VARV-specific transcript B19R, which is deleted in mpox virus (MPXV). This assay specifically detects VARV RNA but not MPXV RNA in respectively infected tissues (Fig 3). Epithelial tissues from the three placebo-treated macaques that did not survive to the end of the study and were positive by IHC were also found to be positive by our newly developed *in situ* hybridization technique designed specifically for variola virus. (Fig 4A and 4B). Additionally, immunofluorescence (IFA) was performed on haired skin of NHP #13 and #4, placebo-treated NHPs that met euthanasia endpoint criteria prior to the end of the study. IFA demonstrates VARV infection primarily infects epithelial cells, but not macrophages (Fig 4C and 4D).

All three surviving placebo-treated animals had histologic lesions within the haired skin consistent with resolving (healing) pox lesions (lymphohistiocytic dermatitis with epidermal hyperplasia [Table 4]), in addition to lymphoid hyperplasia. We were unable to detect poxviral antigen using immunohistochemistry and/or *in situ* hybridization in any of the three placebo-treated survivors at the end of the study (Table 5).

**Table 4:** Summary of histologic findings for placebo and tecovirimat treated NHPs that survived to end of study by tecovirimat treatment group.

Location Histologic Findings	Nonhuman Primate Number														
	Placebo			Day 2 Treatment							Day 4 Treatment				
	7	2	15	11	18	3	8	6	12	1	16	14	17	5	10
**Haired skin** Dermatitis, lymphohistiocytic, with epidermal hyperplasia resolving pox lesion	2	2	2	—	—	—	—	—	—	—	—	—	2	—	—
**Nares** Dermatitis, lymphohistiocytic, with epidermal hyperplasia resolving pox lesion	—	—	—	2*	—	—	—	—	—	—	—	—	—	—	—
**Lip** Dermatitis, lymphohistiocytic, with epidermal hyperplasia resolving pox lesion	—	—	—	—	—	—	—	—	—	—	—	—	—	1*	—
**Tonsil** Follicular hyperplasia		1	1	1	—	1	1	1	1	—	—	1	1	1	1
**Lymph node** Follicular hyperplasia +/- sinus histiocytosis	1	1	1	2	—	1	1	1	1	1	2	1	1	1	1
**Spleen** White pulp hyperplasia	1	2	2	2	1	2	2	2	2	2	2	2	2	1	2

*Unclear if this is related to previous poxviral infection; it may be a nonspecific finding.

Severity scores: 1 = Minimal, 2 = Mild, 3 = Moderate, 4 = Severe;— = no finding

NOTE: No significant lesions were seen in the following organs: tongue; submandibular salivary gland, larynx, esophagus, trachea, thyroid gland, parathyroid gland, testis**, liver, adrenal glands, pituitary gland, kidneys, urinary bladder, prostate gland, heart, lung, skeletal muscle sciatic nerve, brachial plexus, stomach, pylorus, duodenum, jejunum, ileum, cecum, proximal and distal colon, brain, eyes, and femoral bone marrow.

** NHP 14: Testis—inflammation, lymphohistiocytic and eosinophilic, multifocal, mild with fibrosis and spermatogenic arrest

**Table 5:** Immunohistochemistry and in situ hybridization findings: Placebo-treated NHPs.

Location	Nonhuman Primate Number								
	Met clinical criteria and euthanized before end of study						Survived and euthanized at end of study		
	9		13		4		7	2	15
	IHC	ISH	IHC	ISH	IHC	ISH	IHC	IHC	IHC
Haired skin	4+	/	4+	4+	4+	4+	-	-	-
Tongue	4+	4+	-	-	-	-	-	-	-
Tonsil	-	-	4+	4+	4+	4+	-	-	-
Nares	-	-	-	-	1	1	-	-	-
Lip	2+	2+	-	-	4+	4+	-	-	-
Lymph Node(s)	-	-	-	-	-	-	-	-	-
Larynx	2	/	-	/	-	/	-	-	-
Jejunum	-	-	-	-	1	-	-	-	-
Ileocecal Junction	-	-	-	-	1	-	-	-	-
Lung	-	-	-	-	-	-	-	-	-
Spleen	-	-	-	-	-	-	-	-	-

All tissues were stained for IHC; if tissues are not listed, they were negative

/ = ISH data was unavailable

Intensity of staining is graded on the following scale

- = No significant staining

1… .. 1–10 cells/high power field (hpf)

2… .. 11–20 cells/hpf

3… .. 21–40 cells/hpf

4… .. >40 cells/hpf

+ = very strong, positive staining

For animals that were treated with tecovirimat beginning on Day 4, two animals, #14 and #17, had mild inflammation within the haired skin resembling previously described resolving pox lesions, (lymphohistiocytic dermatitis with epidermal hyperplasia) (Table 4). This correlated with the pale, circular areas of skin noted on gross examination. A non-specific finding of unknown clinical significance in a Day 4 treated animal included one primate with mild chronic inflammation in the nares (#5). Similar to the Day 2 treated animals (described in the next paragraph), the Day 4 treated animals exhibited follicular hyperplasia in the tonsil (4/6) and lymph node (6/6), and white pulp hyperplasia in the spleen (6/6), but these animals lacked evidence of active infection with poxvirus using immunohistochemistry, immunofluorescence, *in situ* hybridization, or electron microscopy. In NHP 14 within the testis, there was active production and division of germ cells along the basement membrane of the seminiferous tubules, but the lumina of most seminiferous tubules had few spermatozoa. This indicates an arrest in production of sperm, with a subsequent re-initiation of production. The exact cause is not histologically evident, but systemic infection with VARVand a resultant febrile response followed by convalescence is the most likely explanation for this finding.

For animals that were treated with tecovirimat beginning on Day 2, histologic findings were sparse and most findings were indicative of an immunological response to disease. Histologic findings included follicular hyperplasia in the tonsil (5/6) and lymph node (5/6), and white pulp hyperplasia in the spleen (6/6), findings which are consistent with an appropriate immune response to an infectious agent. Furthermore, these animals lacked evidence of active infection with poxvirus using immunohistochemistry, immunofluorescence, *in situ* hybridization, or electron microscopy. In all groups, pale, raised pleural lesion(s) or pleural adhesions were seen during gross necropsies, but no pathology or associated IHC positivity were seen in any lung tissue of any animals. This points towards a non-specific inflammatory response within all groups.

### Electron microscopy results

**Fig 5:**
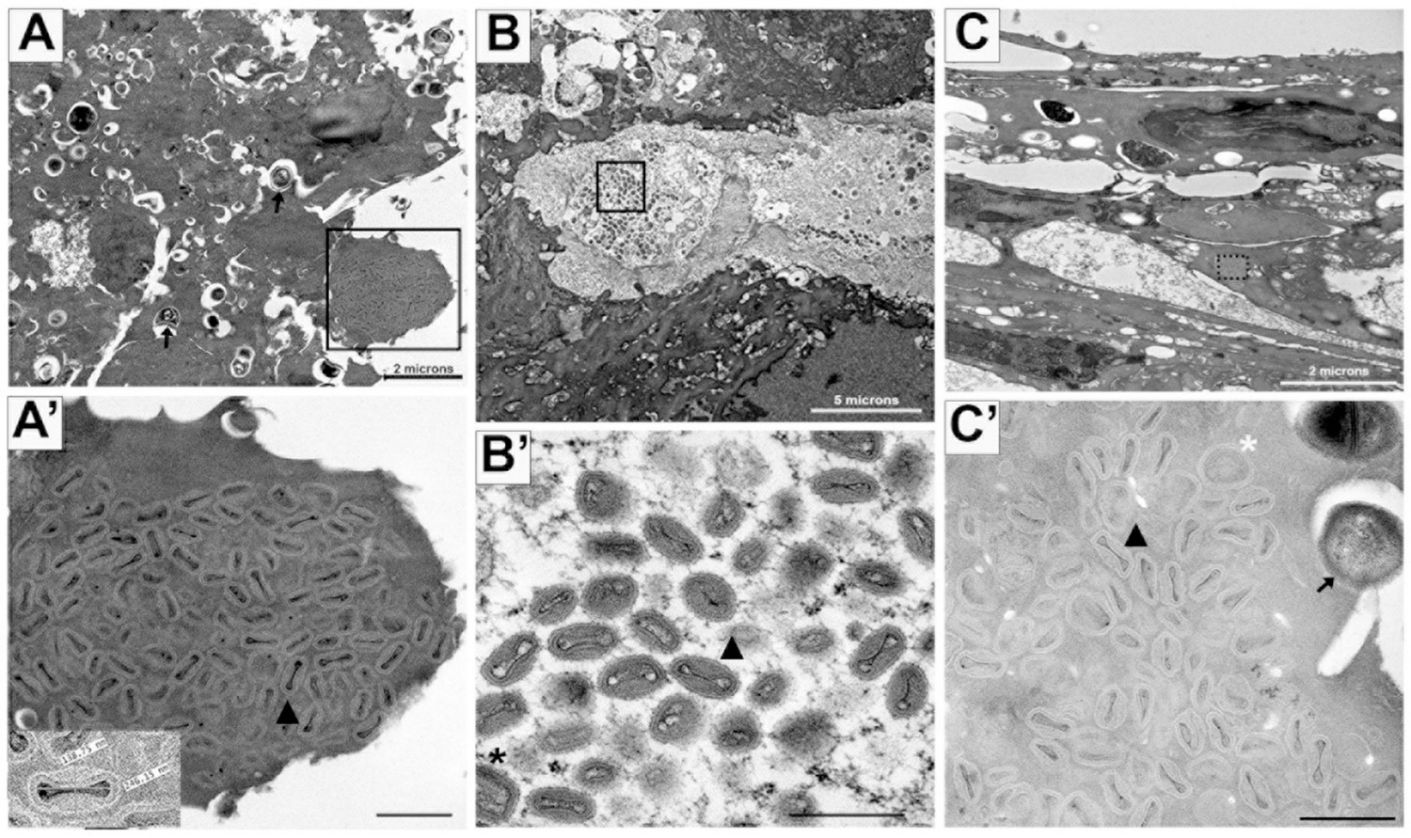
Transmission electron micrographs of VARV infected, placebo-treated NHP haired skin. Panels A, B, C all reveal flaking epidermis and disorganized dermal skin layers in three euthanized variola infected, placebo-treated non-human primates. Panels A’, B’, and C’ are higher magnification images of the boxed regions in panels A, B, and C, respectively, revealing the presence of VARV. Immature (5C’, white asterisk) and mature virions are evident. Brick-shaped, enveloped mature (black asterisk) and mature virions with bar-bell shaped cores (arrowheads) are readily visible. Inset in A’ shows the dimensions (~ 139nm X 246nm) of one mature virion. Superficial bacteria are also evident (arrows) in panels A and C’ (arrows). A’, B’, C’ scalebar = 500nm.

Haired skin samples from four placebo-treated animals and two non-human primates (NHPs) treated 4 days post-infection with tecovirimat were examined. Abundant VARV are present in the haired skin of three of the four placebo-treated controls euthanized for meeting endpoint criteria (Fig 5). All three control samples of haired skin also exhibit superficial bacteria. The virions and bacteria are juxtaposed on the cell surface and within the epithelial skin layers (Fig 5). All three samples display areas of disrupted cell layers with an invasion of immune cells, to include macrophages, and red blood cells. Some regions show an increase in fibrous material surrounding clusters of viral particles. Three phenotypes, (1) circular immature (Fig 5C’, white asterisk), (2) mature virions with bar-bell shaped cores (Fig 5A’, 5B’, 5C’, arrowhead) and (3) bricked shaped envelope mature virions (Fig 5B’, black asterisk), are prominent in the viral clusters (Fig 5). The virions measure, on average, 262nm in length and 142nm in width (See Fig 5A’).

**Fig 6:**
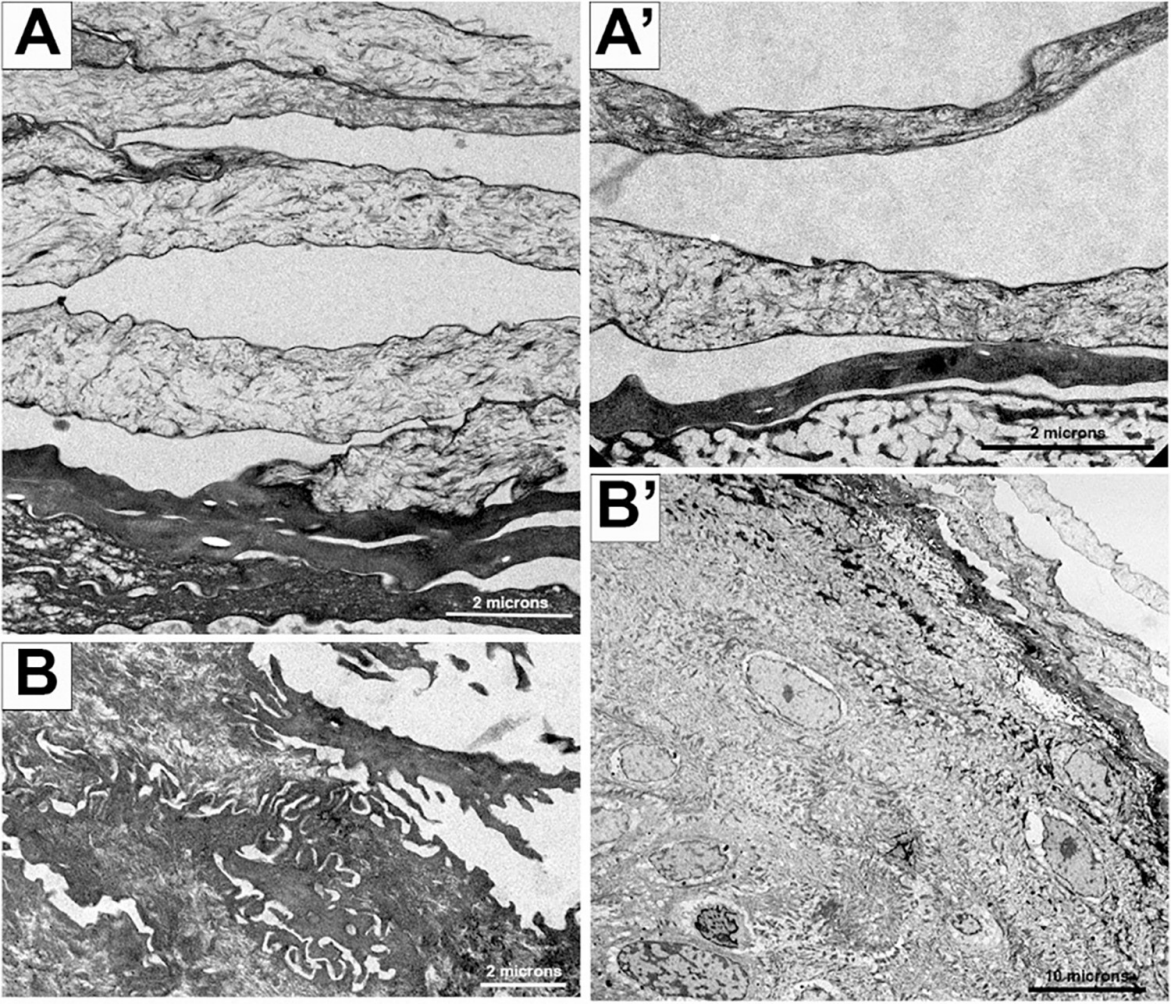
Transmission electron micrographs of VARV infected, tecovirimat-treated NHP haired skin. Panels A and B from surviving NHPs reveal more organized epidermis and dermis skin layers in two end-of-study VARV infected, tecovirimat-treated non-human primates. Panels A’ and B’, are slightly higher magnifications of the epidermis and dermis layers, respectively. No variola virus is evident.

The placebo-treated control that survived to the study’s conclusion, as well as the two animals that were treated 4 days post-exposure, display normal cellular architecture of the haired skin with clearly distinct cell layers. Superficial bacteria are also seen in the surface layers of the haired skin in the infected control animal and one treated survivor. No VARV are seen in any of these samples. Furthermore, an increasing number of stratum corneum layers is seen in all three animals, but no viral particles are present in this expanded layer (Fig 6).

## Discussion

With the 2022 global emergence of MPXV [34–37], there has been renewed interest in research to better understand orthopoxviruses. Recent studies at USAMRIID and the CDC have shown the efficacy of tecovirimat against MPXV and VARV [13,23,38]. Because of the paucity of data regarding smallpox lesions in NHPs, this retrospective pathologic analysis was done to describe the gross, histologic, and ultrastructural smallpox lesions in both placebo-treated and tecovirimat-treated NHPs. A secondary goal of this analysis was to interrogate archived tissues to determine if virus remained in any tissues in placebo-treated or tecovirimat-treated macaques. Newly developed molecular techniques to detect VARV aided in this determination. Recent data has shown viral persistence in immune privileged sites (gonad) in NHPs infected with MPXV [29], and this discovery warranted review of these tissues in smallpox infected NHPs.

In this study, all NHPs were treated daily until day 17 with either the placebo or tecovirimat, and our goal was to describe the differences in the lesions and viral detection in those lesions between groups. In the placebo treatment group, three animals succumbed to disease prior to the end of the study, and all three had gross, histologic, and immunohistochemical findings consistent with an ongoing VARV infection. We were also able to use newly developed ISH probes to detect VARV, and these specific probes have added utility because they can delineate VARV from MPXV in tissues.

The remaining three placebo-treated animals all had gross and histologic lesions consistent with a resolving poxviral infection. There was no detectable VARV signature via IHC, ISH, IFA, or EM in these 3 placebo-treated end-of-study survivors. This suggests viral clearance is complete by day 28 in the target tissues examined. Unlike other high-consequence viruses that have shown evidence of viral persistence following recovery, VARV did not appear in resolving lesions through interrogation with our standard and newly developed assays. The overall number of NHPs in this study was very small, and previous studies done in development of this animal model showed a robust amount of variola antigen within the testes during studies that ended on day 11. [33]. Given the relatively small sample number and how rare this finding could be, it remains to be seen if viral antigen would be seen in the testes and surrounding tissues if the overall number of NHPs was increased with additional studies.

For animals treated with tecovirimat beginning at 4 days post-infection, 2/6 animals had enlarged lymph nodes, 2/6 had a dark, enlarged spleen, and 2/6 had lesions consistent with resolving cutaneous pox lesions similar to placebo-treated survivors. Histologically, 6/6 animals had hyperplasia within the lymph nodes and 6/6 had white pulp hyperplasia within the spleen. Follicular hyperplasia was also present within the tonsils of 4/6 animals. These histologic findings are indicative of antigenic stimulation and are expected in animals mounting an appropriate immune response to an infectious agent. No VARV signature was detected in any animal in this group through IHC, ISH, IFA, or EM. Even when tecovirimat was not administered until after the onset of lesions (day 4 post-infection), the NHPs were able to completely clear all remnants of virus. This was also confirmed via electron microscopy.

In NHPs treated with tecovirimat beginning at 2 days post-infection, or prior to lesion onset, there were no specific gross, histologic, or immunohistochemical findings indicating poxviral infection. Grossly, the enlarged lymph nodes (1/6) and dark, enlarged spleen (3/6) correlated histologically to lymphoid hyperplasia in both of these organs, which is a non-specific response to stimulation from a wide variety of antigens. No variola antigen was detected in the day 2 tecovirimat treatment group via any of our assays in any animal in this group. This indicates treatment with tecovirimat as late as 2 days postinfection results in minimal to no gross or histological lesions, to include no detection of viral protein or viral nucleic acid with the methods described above. The grossly evident pleural adhesions seen across all groups was attributed to non-specific inflammation based on a lack of corresponding histologic findings and a complete absence of IHC positivity in the pleural lining of all examined NHPs.

## Conclusion

Overall, tecovirimat ameliorated the typical gross and histologic lesions associated with VARV induced disease in macaques when animals were treated as late as day 4 post-infection. Placebo-treated animals experienced a 50% mortality rate, with lesions and viral identification as described in previous studies, demonstrating consistency of this animal model. Additional methods (ISH, IFA) that were optimized for smallpox infection assisted in confirmation of viral presence in the affected tissues. These assays help identify viral protein, viral nucleic acid, and cell types infected. These assays also suggested lack of viral persistence in examined tissues.

Initiation of treatment as late as 4 days post infection, representing the post-lesional model, resulted in 100% survival. In the post-lesion treatment model, animals did have some residual pathologic changes similar to the placebo-treated survivors. This indicates that, while the treatment was successful in eliminating mortality in this group and reducing morbidity, the delayed treatment course did result in development of some minor residual lesions. Treatment administered 2 days postinfection not only resulted in 100% survival but had the additional benefit of preventing any significant pathologic response over the duration of the study. The ability to treat as early as possible, in this case prior to lesion development, can have an impact on disease course and residual pathology.

Some of the limitations of this study include a limited number of animals [18], as well as a single-sex cohort (only males were utilized in these studies). While we did not see any viral persistence due to the limited number of animals in this study, further persistence research should be explored. Viral antigen was found in testes in the Wahl-Jensen study [33], and a larger number of subjects may have resulted in finding virus in the testes of some NHPs in this study.

Mpox, an orthopoxvirus closely related to VARV, has shed a spotlight on the gap in our collective immunity to orthopoxvirues. Understanding the molecular mechanisms of disease for these viruses is of paramount importance. Given the highly transmissible nature of these viruses, any re-emergence of smallpox in now unvaccinated populations is likely to lead to widespread infection before the diagnosis of the index case. Therefore, a better understanding of the pathogenesis of this disease with development of a robust intervention strategy, including the optimization of existing United States Food and Drug Administration (FDA) licensed vaccines (ACAM2000, JYNNEOS), novel therapeutics, and other public health measures, is of utmost importance.

## Materials and methods

### Ethics statement

All animal studies were conducted in compliance with the Animal Welfare Act and other federal statutes and regulations relating to animals and experiments involving animals and adheres to principles stated in the Guide for the Care and Use of Laboratory Animals, National Research Council. All animal experimental protocols were approved by a preexisting internal institutional animal care and use committee (IACUC). The facilities where this research was conducted are fully accredited by the Association for Assessment and Accreditation of Laboratory Animal Care International. Animals meeting criteria were humanely euthanized.

### Animals, exposure, and treatment

Details about the design, conduct and outcomes of the animals utilized within this manuscript have been previously published by Mucker et al.(23). Briefly, a double-blinded study using 18 male poxvirus naïve Mauritius cynomolgus macaques having age and weight ranging from 2.9 to 6.5 years and 4.8–7.1 kg, respectively, were randomized into two tecovirimat treatment groups and a single placebo control group. All animals were dosed with either placebo or a combination of placebo and tecovirimat for 16 consecutive days (days 2–17). More specifically, animals in the tecovirimat treated groups either received drug on days 2–15 or days 4–17, with placebo administration on days 16 and 17, or 2 and 3, respectively. Animals in the control group received placebo from days 2–17. On Study Day 0, NHPs were intravenously infected with 1 x 10^8^ pfu of Harper strain of VARV in one mL volume.

The overall goal of these studies was to evaluate the post-exposure prophylactic (pre-lesion/rash onset) and therapeutic (post-lesion/rash onset) efficacy of tecovirimat against VARV using the intravenous lesional model of smallpox in nonhuman primates (NHPs). In this model, skin and/or mucosal lesions/rash are typically observed three to five days after exposure. Treatments were delayed by either two (pre-lesion onset) or four (post-lesion onset for most animals) days post-exposure to assess tecovirimat for post exposure prophylaxis and therapeutic indications. Animals were orally administered placebo or tecovirimat and/or placebo daily from Days 2–17 to maintain blinding. More specifically, NHPs received 10 mg/kg/day of tecovirimat for a total of fourteen days, with placebo given two days prior to initiation (Day 2 treated group), or two days after completion of the tecovirimat regimen (23). The primary endpoint was total lesion count due to smallpox disease. Other experimental endpoints included viral loads (based on qPCR [39]), lesion onset and progression, and survival.

Volume dosing for both test and control articles (e.g., tecovirimat or placebo) were calculated on individual weights of animals at the initiation of the study. All experiments and procedures involving VARV were performed at the Center for Disease Control and Prevention (CDC). Formalin-fixed, paraffin-embedded blocks from this study are stored at USAMRIID in a temperature and humidity-controlled environment.

### Gross necropsy

A full necropsy was performed on all animals and recorded by a board-certified veterinary pathologist. A summary of animal numbers, pathology accession numbers, challenge dates, and necropsy dates is presented in Table 1. The following tissues were collected at necropsy and immediately immersed in 10% neutral buffered formalin (NBF): skin (with, if present, and without pox lesions), tongue; tonsil, lip, nares, mandibular, tracheobronchial, axillary, inguinal, and mesenteric lymph nodes, submandibular salivary gland, larynx, esophagus, trachea, thyroid gland, parathyroid gland, testis, liver, spleen, adrenal glands, pituitary gland, kidneys, urinary bladder, prostate gland, heart, lung, skeletal muscle sciatic nerve, brachial plexus, stomach, pylorus, duodenum, jejunum, ileum, cecum, proximal and distal colon, brain, eyes, and femoral bone marrow. Tissues were fixed for a minimum of 21 days in 10% neutral buffered formalin. The in-life portion of this study was completed at the CDC and, after viral inactivation via formalin fixation, FFPE tissues were analyzed by the pathology team at USAMRIID, Fort Detrick, Maryland.

### Histopathology

The tissues were trimmed and processed according to standard protocol. Sections were trimmed to 5–6 μm and routinely stained with hematoxylin and eosin. Replicate sections were processed for immunohistochemistry (IHC), *in situ* hybridization (ISH) and immunofluorescence (IFA). A board-certified veterinary pathologist evaluated the H&E, IHC, and ISH by light microscopy. IFA was evaluated by a molecular biologist utilizing confocal microscopy. Results for each animal are summarized in Tables 5–9.

### Immunohistochemistry

Immunohistochemistry (IHC) was performed on replicate tissue sections using an Envision kit (Dako Agilent Pathology Solutions). An in-house polyclonal antibody against vaccinia virus (#1293, USAMRIID) was used at a dilution of 1:3500. After deparaffinization, rehydration, methanol-hydrogen peroxide blocking, and decrosslinking using higher-pH antigen retrieval buffer, sections were covered with primary antibody and incubated at room temperature for sixty minutes. They were rinsed, and then the peroxidase-labeled polymer (secondary antibody) was applied for thirty minutes. Slides were rinsed and a substrate-chromogen solution was applied for 5 minutes. All slides were exposed to brown chromogenic substrate, 3,3’-diaminobenzidine (DAB; Dako Agilent Pathology Solutions), counterstained with hematoxylin, dehydrated, and coverslipped.

### In Situ hybridization

*In situ* hybridization was performed on haired skin and mucosal epithelium. To detect smallpox viral messenger RNA (mRNA) in FFPE tissues, ISH was performed using the RNAscope 2.5 HD Detection Kit (RED) for FFPE Tissues (Advanced Cell Diagnostics, Newark, CA, USA) according to the manufacturer’s instructions. Briefly, an ISH probe targeting fragment 2–1311 of smallpox virus-specific gene B19R [13], which aligns to 165795–167421 of LT706528.1., was designed and synthesized by Advanced Cell Diagnostics (Cat# 534681) and an ISH probe targeting MPXV-specific gene D1L was reported previously (Cat#534671, Advanced Cell Diagnostics). Tissue sections were deparaffinized with Xyless II (Val Tech Diagnostics, Brackenridge, PA, USA), underwent a series of ethanol washes and were peroxidase blocking, heated in kit-provided antigen retrieval buffer, and then digested by kit-provided proteinase. Sections were exposed to ISH target probe pairs and incubated at 40°C in a hybridization oven for 2 h. After rinsing, ISH signal was amplified using kit-provided pre-amplifier and amplifier conjugated to alkaline phosphatase and incubated with a Fast Red substrate solution for 10 min at room temperature. Sections were then stained with hematoxylin, air-dried, and coverslipped.

### Immunofluorescence

Immunofluorescence was performed on haired skin, and mucosal epithelium. Formalin-fixed paraffin embedded tissue sections were deparaffinized using xylene and a series of ethanol washes. The sections were heated in Tris-EDTA buffer (10mM Tris Base, 1mM EDTA Solution, 0.05% Tween 20, pH 9.0) for 20 minutes to reverse formaldehyde crosslinks. After rinses with phosphate-buffered saline (PBS), pH 7.4 (Thermo Fisher Scientific, Walthan, MA, USA), sections were blocked overnight with PBT (PBS + 0.1% Tween-20) containing 5% normal goat serum (Millipore Sigma, Burlington, MA, USA) at 4°C. Sections were then incubated with the following primary antibodies for 2 h at room temperature as follows: rabbit polyclonal antibody (USAMRIID, Frederick, MD USA) against vaccinia virus, which cross binds to smallpox virus, at a dilution of 1:500 and mouse monoclonal anti-human CD68 antibody (Clone KP1; Dako Agilent Pathology Solutions) at a dilution of 1:200 or mouse monoclonal anti-Cytokeratin 10 antibody (ab20208; Abcam, Cambridge, MA, USA) at a dilution of 1:100. After rinsing in PBT, sections were incubated with secondary goat Alexa Fluor 488-conjugated anti-rabbit and with goat Alexa Fluor 561-conjugated anti-mouse antibody for 1 h at room temperature. Sections were coverslipped using VECTASHIELD antifade mounting medium with DAPI (Vector Laboratories, Burlingame, CA, USA). Images were captured on a LSM 880 Confocal Microscope (Zeiss, Oberkochen, Germany) and processed using open-source ImageJ software (National Institutes of Health, Bethesda, MD, USA).

### Electron microscopy

Three 1 mm tissue punches were made from the six tissue blocks provided. Samples were heated for at 60°C for 45 minutes and deparaffinized in Xyless II for 30 minutes. The 18 tissue plugs were rehydrated in 100% ethanol for 15 minutes and then 95% and 70% ethanol before being fixed in 4% paraformaldehyde and 1% glutaraldehyde and 0.1M sodium cacodylate buffer for 1hour. Samples were subsequently rinsed with 0.2M sucrose in buffer for 30 minutes then post-fixed for 1% OsO4 for 1 hour and finally rinsed in buffer again for 30 minutes. Samples were dehydrated with increasing concentration of ethanol (50%, 75%, 95%, and 100%) for 10 minutes each. The samples were further dehydrated in 100% propylene oxide (PO) and 100% ethanol [1:1], followed by two changes of 100% PO for 10 minutes. Samples were then infiltrated with 100% PO and epoxy resin [1:1] then [1:2] overnight. Samples were incubated in 2 exchanges of pure epoxy resin before being embedded, oriented and polymerized at 60°C for 48 hours.

Five hundred-nanometer sections were cut and counterstained with toluidine blue from all 18 samples. Toluidine blue stained images verified tissue integrity and served as a guide for TEM imaging. 75–85 nm thin sections were cut of these regions, collected on 200 mesh copper grids and post-section stained with uranyl acetate and Reynold’s lead citrate. Grids were imaged on the JEOL TEM1400 at various magnifications using the Gatan Rio9 camera.
